# The microstructure in the placenta is influenced by the functional diversity of HLA-G allelic variants

**DOI:** 10.1007/s00251-019-01121-0

**Published:** 2019-06-27

**Authors:** F. Stieglitz, A. A. Celik, C. von Kaisenberg, M. A. Camps, R. Blasczyk, Christina Bade-Döding

**Affiliations:** 1 Hannover Medical School, Institute for Transfusion Medicine, Carl-Neuberg-Str. 1, 30625 Hannover, Germany; 2 Department of Obstetrics and Gynecology, Hannover Medical School, Carl-Neuberg-Str. 1, 30625 Hannover, Germany; 3 Imusyn GmbH & Co. KG, Feodor-Lynen-Str. 5, 30625 Hannover, Germany

**Keywords:** dNK cells, HLA-G, Placenta, Development, Decidua, Pregnancy

## Abstract

The main expression sites of HLA-G are human extravillous trophoblast cells. The interaction of HLA-G with uterine NK cells promotes their maturation and differentiation into decidual NK (dNK) cells. dNK cells secrete chemokines, cytokines, and proangiogenic factors in favor of a vascular remodeling and an immune suppressive microenvironment of the decidua. HLA-G is the most polymorphic member of the oligomorphic non-classical HLA molecule family; yet, the impact of polymorphic differences is not comprehensively understood. sHLA-G levels in embryo culture medium correlate with successful pregnancy; however, it remains questionable if HLA-G allelic diversity impacts on the outcome of dNK cell development. We utilized synthetic sHLA-G*01:01, *01:03*, and *01:04* molecules and transduced K652/mHLA-G*01:01, *01:03*, and *01:04* cells to study the biological interaction between HLA-G alleles and primary NK cells of human term placenta. Despite its low frequency, HLA-G*01:04 and not the most prevalent allele HLA-G*01:01 appear to be strong catalysts of dNK cell proliferation. Concluding, this study illustrates novel insights into the impact and binding efficiency of the three most common variants of HLA-G on primary placental NK cells.

**Electronic supplementary material:**

The online version of this article (10.1007/s00251-019-01121-0) contains supplementary material, which is available to authorized users.

## Introduction

HLA-G, a non-classical HLA-I molecule, features specialized abilities to confer immune protection by, e.g., suppressing the immune system, promoting vascularization of the decidua, and promoting tolerance induction of the maternal immune system towards the fetus. Due to its oligomorphic nature, HLA-G has only 58 allotypes (Robinson et al. 2014) with HLA-G*01:01 predominantly expressed followed by G*01:03 and G*01:04. The most recent study revealed that those alleles differ highly in their presented peptide repertoire and their mediated protection against NK cell–mediated lysis (Celik et al. 2018a). Few polymorphic variants and their variability in the presented peptide repertoire and allele-specific interaction with NK cells have been described (Celik et al. 2018a). Findings that the sHLA-G level in embryo culture medium correlates with successful pregnancy underline the major importance of HLA-G during gestation (Lynge Nilsson et al. 2014). Considering the significant role of HLA-G in successful pregnancies, the HLA-G allele-specific manipulation of pNK cell function, and the observation that the interaction between HLA-G and its interaction with decidual NK cells (dNK) appears to be vital for implantation, subsidization, and tolerance of the fetus, there might be a potential impact of allelic HLA-G variants. Pregnancies are outstanding events that resemble a substantial challenge to the maternal immune system. The fetus represents a semiallogeneic transplant that has to be protected from rejection by the mother. Under healthy physiological conditions, the placental extravillous trophoblasts (EVTs) are the main expressing site of HLA-G1 and HLA-G5 (Fournel et al. 2000; Juch et al. 2012). Several membrane-bound (G1–G4) and soluble (G5–G7) isoforms that are varying in the composition of their α subunits exist and are generated by alternative splicing. Although for those isoforms receptors are suggested, it remains unclear whether they have a significant function in vivo (Toni Ho et al. 2018). During the process of implantation, fetal trophoblast cells invade the maternal decidua and the spiral arteries, creating large vessels with a lower resistance for the circulating maternal blood; this increases the blood stream to the fetus (Lyall et al. 2001).

dNK cells play a pivotal role in the process of trophoblast invasion. Peripheral NK (pNK) cells represent < 15% of the lymphocyte population (Timonen and Ortaldo 1981), while the NK cell population of the decidua basalis represents approximately 70% of lymphocytes in the early stage of pregnancy (King et al. 1989; Moffett-King 2002). The main task for pNK cells is to kill maligned and virus-infected cells (Jost and Altfeld 2013); in contrast to that, dNK cells lost their cytotoxic abilities under healthy conditions completely (Kopcow et al. 2005) and further promoting an immunosuppressive status by stimulating the production and release of indoleamine 2,3-dioxygenase (IDO) by dCD14^+^ cells leading to the induction of Tregs (Vacca et al. 2010). Noteworthy, KIR (KIR3DL1, KIR3DL2, KIR2DL3, and KIR2DL4) receptors are significantly upregulated in dNK cells compared with pNK cells; interestingly, the KIR2DL4 receptor is only present in dNK cells, indicating an adaptation to the presence of HLA-G (Koopman et al. 2003). Moreover, dNK cells promote decidual vascularization and concentrate nearby the spiral arteries while releasing MMPs that lead to the maceration of extracellular matrix of the vascular smooth muscle cells. This process promotes the vascular remodeling that is vital for the supplementation of the fetus (Hazan et al. 2010; Robson et al. 2012; Smith et al. 2009). To conclude, dNK cells distinguish significantly from pNK cells in terms of cytotoxicity, release of cytokines and chemokines, their immune modulatory properties, and their promoting of vascular growth (Le Bouteiller 2013). Notably, low quantities of dNK cells are highly associated with recurrent spontaneous abortions (Quack et al. 2001).

The origin and development of dNK cells is still not comprehensively investigated, yet two theories attempt to explain their origin (Jabrane-Ferrat and Siewiera 2014). One theory describes that dNK cells are derived from local progenitor cells or immature uterine NK (uNK) cells. This theory is based on the observation that dNK cells could be developed from progenitor cells in situ by a certain chemokine/cytokine and hormone cocktail (Kane et al. 2009; Vacca et al. 2011). Furthermore, local immature uNK cells could be found in the uterus and regulate the development cycle of the endometrium during the menstruation cycle and their count shifts along with the IL-15 concentration. CD34^+^ NK lineage precursor cells that could be detected in the uterus differentiate to dNK cells in the presence of IL-15 (Bilinski et al. 2008; Manaster and Mandelboim 2008).

The second theory assumes that CD56^bright^ pNK cells migrate from the periphery into the decidua where they are re-educated and develop into dNK cells in the decidual microenvironment (Jabrane-Ferrat and Siewiera 2014). This theory is supported by the observation that EVT cells produce large amounts of NK-specific chemokines and that pNK cells could be primed to develop a dNK phenotype utilizing TGF-β or IL-15 (Carlino et al. 2008; Cerdeira et al. 2013; Drake et al. 2001; Hanna et al. 2003; Kunkel and Butcher 2002).

In the last decade, HLA-G emerged as an additional key factor for dNK cell development and maintenance of the dNK cell status and continuous proliferation. It could be demonstrated that uNK cells begin to proliferate and secrete INF-γ and VEGF during co-incubation with cells expressing transduced membrane-bound HLA-G (mHLA-G). This experiment was performed with human uNK cells before conception, indicating that the contact of uNK cells with mHLA-G on EVTs could lead to the maturation of uNK cells to dNK cells. dNK cells promote vascularization and decidualization of the implantation site; this effect was not seen when the whole uterine mononuclear cell population (UMC) was incubated with mHLA-G expressing cells (van der Meer et al. 2004). Besides, it could be demonstrated that the acquirement of mHLA-G from the cell surface of EVTs via trogocytosis by dNK cells is essential for maintaining their low-level cytotoxicity (Tilburgs et al. 2015).

In the light of recent attempts to generate dNK cells in vitro (Cerdeira et al. 2013) with the overall aim to treat reproductive disorders, the importance of understanding the impact of HLA-G subtypes on the maintenance and development of the dNK cell population becomes obvious.

## Material and methods

### Cell lines

All cell lines were maintained at 37 °C and 5% CO_2_. HLA class I negative *K562* cells transduced with HLA-G*01:0x (exons 1–6) (Celik et al. 2018a) or sHLA-G*01:0x (soluble, exons 1–4) (Celik et al. 2018a; Celik et al. 2018b), and NK cell cultures were maintained in RPMI1640 (Lonza, Basel, Switzerland) supplemented with 10% heat-inactivated FCS (Lonza, Basel, Switzerland), 2 mM L-glutamine (c. c. pro, Oberdorla, Germany), 100 U/mL penicillin and 100 μg/mL streptomycin (c. c. pro, Oberdorla, Germany), and 1.8 × 10^3^ U/mL ProLeukinS® (Novartis, Basel, Switzerland).

*HEK293T* cells, used for production of lentiviral particles, were cultured in DMEM (Lonza, Basel, Switzerland) supplemented with 10% heat-inactivated FCS, 2 mM L-glutamine, 100 U/mL penicillin, 100 μg/mL streptomycin, and 1 mg/mL Geneticin® (Life Technologies, Carlsbad, USA). All cell lines were maintained at 37 °C and 5% CO_2_.

### Isolating NK cells from term placental tissue

dNK were isolated from a whole human placenta. Human term placenta was obtained from the cesarean section at the term of pregnancy. The obtained term placentas weigh about 750–1250 g; 250 mL PBS containing 50 U/mL Na/heparin was added immediately after cesarean section to the placenta at RT. Subsequently, the placenta was minced with sterile-disposable scalpels into 2 × 2 cm large pieces. Those pieces were transferred to a petri dish, crumpled with the hard end of a 60-mL syringe plunger, and then rinsed with 5 mL PBS/heparin solution; the tissue was discarded, and the flow through was collected. After processing the whole placental tissue, the flow through was centrifuged at 422*×g* for 10 min; the resulting pellets were pooled in 25 mL PBS and separated twice by density gradient centrifugation utilizing *Lymphosep™* medium. Between the isolation steps, the collected lymphocytes were washed once with 25 mL PBS (270*×g*, 10 min) to remove uptaken *Lymphosep™* medium (MP Biomedicals, LLC, OH, USA). After density separation, cells were washed twice with 25 mL PBS (270*×g*, 10 min; 187 g, 10 min). Finally, cells were resuspended in 10-mL placenta medium per 1 × 10^8^ cells. Isolation of CD56^+^ from isolated placental cells has been performed using the *EasySep™ Human NK Cell Enrichment Kit* (Stemcell Technologies, Vancouver, Canada).

### HLA-G genotyping

**Fig. 1:**
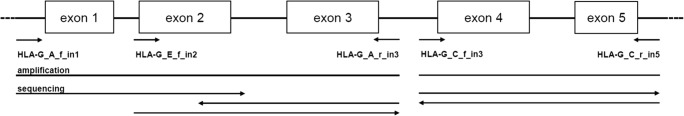
Location of HLA-G primers for amplification and sequencing

To determine the HLA-G alleles of the utilized placenta, donor DNA was isolated utilizing the *NucleoSpin® Blood* kit (Macherey-Nagel, Düren, Germany). Specific primers were used to generate the required parts (Fig. 1) of the donor DNA for genotyping (Table 1). PCR solution contains 1.25 μL forward and reverse primer (2.5), 2 μL DNA, 5 μL betaine (5 M), 12.5 μL Bio-X-Act, and 3 μL H_2_0. Total volume was 25 μL. Following amplification, the PCR products were purified using *EXOSAP-IT* (Thermo Fisher Scientific, Waltham, USA) and subjected to Sanger sequencing. Sequence analysis was performed by HiType (Inno-Train Diagnostics, Kronberg, Germany) using the most recent HLA database release.

**Table 1:** Primer for HLA-G genotyping

Name	Direction	Sequence
HLA-G_A_f_in1 (FWD)	Forward	5′-tctaaagtcctcgctcacc-3′
HLA-G_A_r_in3 (REV)	Reverse	5′-cttgtgctaggccaggc-3′
HLA-G_E_f_in2 (FWD)	Forward	5′-cctcttcctgctgctctc-3′
HLA-G_C_f_in3 (FWD)	Forward	5′-gtcacatccaggtgctg-3′
HLA-G_C_r_in5 (REV)	Reverse	5′-agtgggacaagaaaactcagac-3′

### Production of transduced K562 cells expressing sHLA-G or mHLA-G molecules

Transduced *K562* cells expressing sHLA-G*01:01, *01:03*, or *01:04* have been cultured; sHLA-G*01:0x molecules have been isolated as previously described (Celik et al. 2018a), and transduced *K562* cells expressing mHLA-G*01:01, *01:03*, or *01:04* have been sorted for 100% mHLA-G*01:0x surface expression as described (Celik et al. 2018a).

### dNK cell proliferation assay

To determine the influence of the HLA-G variants on proliferation of decidual NK cells, a proliferation assay was performed as described by van der Meer et al. (2004), with the modification that CFSE dilution was utilized for measuring proliferation. NK cells were labeled with CFSE and plated at 5 × 10^4^ cells/well in triplicates in 96-well U-bottom microtiter plates in the presence of irradiated (30 Gy) *K562* or transduced mHLA-G*01/0x expressing *K562* cells (5 × 10^4^ cells/well) in a total volume of 200 μL. Medium exchange was performed every 3 days. Proliferation analysis was performed at day 6 measuring CFSE dilution of CD56^+^ cells.

### sHLA-G-CD56^+^/CD9^+^ NK cell binding assay

To determine binding potential and variability of the three allelic subtypes, sHLA-G*01:01, *01:03*, or *01:04*, to bind to dNK cells, 5 × 10^6^ dNK cells have been stained for 4 h with purified transduced C-terminally V5-tagged sHLA-G*01:0x molecules at a concentration of 30 μg/mL. Binding was detected using 5 μL anti-V5 (clone MCA1360) (Bio-Rad Laboratories, Inc., Hercules, USA) and goat anti-mouse PE (BD Biosciences, Heidelberg, Germany). Following incubation, 5 μL anti-CD9 FITC (clone eBioSN4) (Life Technologies™, Carlsbad, USA) and 5 μL anti-human CD56-APC (clone NCAM16.2) (BD Biosciences, Heidelberg, Germany) were added to stain the dNK cells. Cell populations were analyzed by flow cytometry.

### Statistical analyses

For statistical analyses of the proliferation data, a one-way ANOVA and Tukey’s multiple comparison test were utilized.

### Ethical approval

Written informed consent was obtained from the patients to donate the placenta to research; ethical committee approval had been obtained (MHH Research Obstetrics Biobank No. 1303-2012).

## Results

### sHLA-G*01:0x binding to CD56^+bright^/CD9^+^ NK cells

**Fig. 2:**
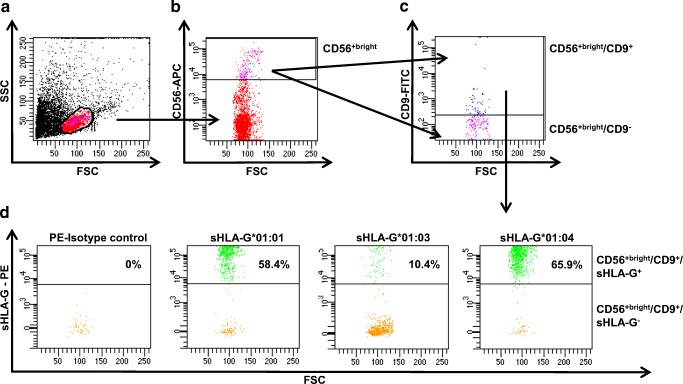
Binding of dNK cells to sHLA-G*01:01, 01:03, or 01:04. Gating strategy (**a**–**c**) and analysis of sHLA-G binding to CD56^+bright^/CD9^+^ cells. **d** sHLA-G*01:01 and saHLA-G*01:04 bind with an overall higher efficiency to the CD56^+bright^/CD9^+^ subset than sHLA-G*01:03. The HLA-G genotype of the represented donor was HLA-G*01:01

To examine the binding efficiency of the three most common HLA-G alleles (G*01:01, G*01:03, G*01:04) to primary dNK cells, purified sHLA-G*01:0x variants were incubated with isolated lymphocytes from human term placenta. It could be observed that sHLA-G*01:01 and sHLA-G*01:04 bind with an overall higher efficiency to the CD56^+bright^/CD9^+^ dNK subset than sHLA-G*01:03 (*01:01* 58.4%; *01:03* 10.4%; *01:04* 65.9%) (Fig. 2). The sHLA-G binding to CD56^+bright^/CD9^+^ NK cells from the same batch varied between 17.8% for 01:01, 1.5% for 01:03, and 20.6% for 01:04 (Suppl. Fig. 1).

### Proliferation assay of primary NK cells from placental tissue

**Fig. 3:**
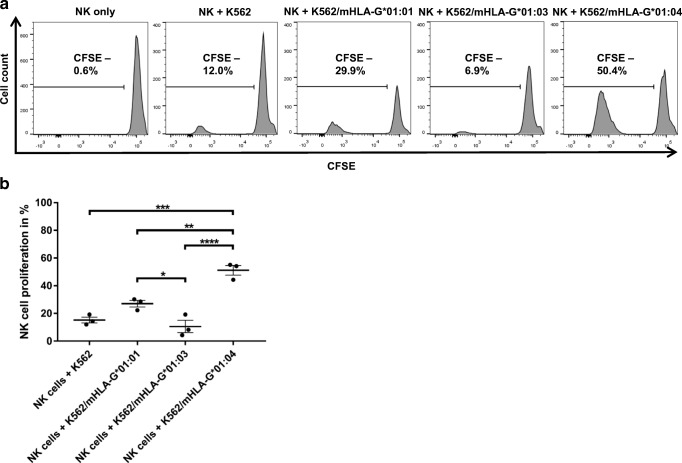
Proliferation of primary dNK cells from human term placenta after exposure to different HLA-G allelic variants. Analysis of proliferation of primary dNK cells from human term placenta after co-incubation with HLA-G*01:01, 01:03, or 01:04 expressing *K562* cells (**a**). Stimulation of proliferation could be unambiguously attributed to HLA-G*01:04. A significant difference in terms of proliferation activation could be detected between mHLA-G*01:01 and mHLA-G*01:03 and between mHLA-G*01:01 and mHLA-G*01:04 (*p* < 0.05; *p* < 0.005) (**b**). ****p value < 0.0001; ***p value < 0.0005; **p value < 0.005; *p value < 0.05

The HLA-deficient cell line *K562* was transduced with lentiviral vectors encoding for mHLA-G*01:01, mHLA-G*01:03, or mHLA-G*01:04 and subsequently sorted for 100% of the respective mHLA-G expression. Untouched primary CD56^+^ NK cells were recovered from human term placenta, stained with CFSE, and co-incubated with the transduced *K562* cells expressing HLA-G for 6 days. Proliferation of NK cells was monitored by CSFE dilution (Fig. 3a). Primary dNK cells incubated with *K562* cells showed 12% proliferation after 6 days. dNK cells that were incubated with mHLA-G*01:04 expressing *K562* cells showed the highest proliferation rate with ~ 50%; NK cells co-incubated with mHLA-G*01:01 or mHLA-G*01:03 expressing *K562* cells had a substantially lower proliferation rate (HLA-G*01:01 ~ 30%; HLA-G*01:03 ~ 7%). The proliferation levels of non-dNK cells from the same batch varied from ~ 12% for 01:01, ~ 4% for 01:03, and ~ 13% for 01:04 (Suppl. Fig. 2). This experiment was performed with three different donors varying in their genotype (donor A HLA-G*01:01/01:05N; donor B HLA-G*01:01/01:01; donor C HLA-G*01:01/01:04:02).

A significant increase in proliferation could be detected when primary NK cells were incubated with K562/mHLA-G*01:04 cells in comparison with *K562* cells or K562/mHLA-G*01:03 cells, respectively (*p* = 0.0002; *p* < 0.0001). Furthermore, a significant difference could be detected between K562/mHLA-G*01:01 and K562/mHLA-G*01:03 and between K562/mHLA-G*01:01 and K562/mHLA-G*01:04 (*p* = 0.0295; *p* = 0.0033) (Fig. 3b).

## Discussion

The immunosuppressive abilities of HLA-G have been extensively studied and recognized in recent years. HLA-G is the most polymorphic representative of the oligomorphic HLA-Ib molecules; however, little is known about the differential impact of the most prevalent allelic variants HLA-G*01:01, HLA-G*01:03, and HLA-G*01:04. We recently identified a remarkable difference between these variants in terms of the presented peptide repertoire and their capability of inhibiting NK cell–mediated lysis, whereby HLA-G*01:04 has been found to convey the most protective status for NK cell–mediated lysis (Celik et al. 2018a). The main function of HLA-G in vivo is to convey an immunotolerant status to the fetus and promote vascularization of the decidua as well as promoting the implantation of the zygote (Ferreira et al. 2017). Therefore, this study concentrated on the functional differences of the most common HLA-G variants and their allelic impact on the proliferation of primary dNK cells from human term placenta.

To analyze binding efficiency and variability of HLA-G variants to primary lymphocytes from human term placenta, the allelic subtypes HLA-G*01:01, *01:03*, or *01:04* were engineered with a C-terminal V5 tag to enable the detection of binding to the subset of primary dNK cells. Notably, sHLA-G*01:04 and sHLA-G*01:01 bound with an efficiency of 65.9% and 58.4%, respectively, whereas sHLA-G*01:03 featured only 10.4% binding efficiency (Fig. 1). Interestingly, Celik et al. (2018) previously described mHLA-G*01:04 to convey the strongest protection against NK cell–mediated lysis (Celik et al. 2018a). This data stands in coherency with the highest binding efficiency observed with sHLA-G*01:04 in comparison with the other allelic variants in this study. Furthermore, no major functional difference between HLA-G*01:01 and HLA-G*01:03 in terms of NK-mediated lysis could be detected (Celik et al. 2018a), while in the present study where primary dNK cells have been applied, a difference of approx. 45% in binding efficiency between HLA-G*01:01 and HLA-G*01:03 could be observed.

**Fig. 4:**
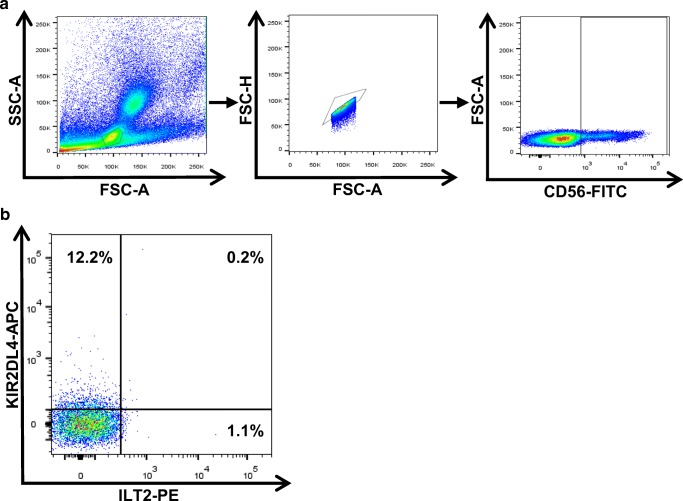
Expression of KIR2DL4 and ILT2 on primary NK cells from human term placenta. Expression levels of KIR2DL4 and ILT2 on freshly isolated NK cells from human term placenta (**b**) and gating strategy (**a**)

Possible explanations for this divergence could be (i) the ability of HLA-G to adapt to the cells where it is expressed (Celik et al. 2018b) and the associated immune functions and/or (ii) a difference in the interacting receptors on the respective immune cells. For inhibiting NK-mediated lysis, ILT2 is the responsible receptor and interaction partner of HLA-G (Shiroishi et al. 2003; Shiroishi et al. 2006). The ILT2/HLA-G interaction is proposed to be mediated mainly through the α3 domain of the HLA-G molecule, although a recent study proposed that the α1 and α2 domains could have also an influence on the ILT2/HLA-G interaction (Nam et al. 2013; Shiroishi et al. 2006). Crystallization studies of KIR2DL2 with HLA-C advocated that KIR binding to HLA molecules is mediated through the α1 and α2 units and the peptide on positions P7 and P8 (Boyington et al. 2000). Based on this crystallization study, an equivalent interaction site for KIR2DL4 could be assumed for HLA-G. Still, the concept of HLA-G being a ligand for KIR2DL4 remains highly controversial since a new crystallization study finds no evidence of KIR2DL4 binding HLA-G or other HLA molecules (Moradi et al. 2015). Additionally, the KIR2DL4 receptor misses the D1–D2 arrangement that is proposed to interact with the α1 and α2 domains (Boyington et al. 2000). Despite those findings, it remains evident that only sHLA-G binding could be observed in this study when KIR2DL4 is present on the cell surface indicating that the presence of KIR2DL4 is necessary for binding. Furthermore, the difference in binding of the allelic variants could only be explained by the differences between the three allelic HLA-G variants that cumulate in AA exchanges in the α1 and α2 domains and an altered peptide repertoire (Celik et al. 2018a; Robinson et al. 2014), indicating a KIR family receptor involvement in the form of KIR2DL4 or yet an unknown KIR receptor that is co-expressed with KIR2DL4. Beyond this, it can be proposed that while the interaction of HLA-G with ILT2 is mainly mediated through the α3 domain and only partially influenced by the α2 domain, the binding of the secondary described receptor KIR2DL4 is assumed to be predominantly determined by the α1 and α2 domains and the presented peptide (Figs. 3 and 4). A peptide-mediated alteration of the overall HLA-G structure could presumably lead to a stronger divergence in binding efficiency, and this could be a possible explanation for the differences observed in NK-mediated lysis (Celik et al. 2018a) versus HLA-G/dNK cell interaction. The presence of an unknown receptor seems to be obvious since the expression levels of KIR2DL4 and ILT2 on the used primary dNK cells revealed expression levels of ~ 12% and ~ 2%, respectively (Fig. 4).

It is proposed that uNK cells start proliferating when incubated with recombinant mHLA-G expressing cells (van der Meer et al. 2004). In the present investigation, the magnitude of allelic HLA-G subtypes on dNK cell proliferation and a possible developmental switch from CD56^+bright^/CD9^−^ to CD56^+bright^/CD9^+^ could be identified. The HLA-G allele-dependent proliferative capacity of NK cells from primary placental lymphocytes stands in coherency with the results of the binding efficiency of the allelic sHLA-G subtypes. Proliferation was significantly higher when primary placental NK cells were incubated with K562/mHLA-G*01:04 compared with the other alleles or parental *K562* cells. Additionally, mHLA-G*01:01 promotes a significant stronger proliferation than mHLA-G*01:03. Furthermore, proliferation is unaffected by the donor’s HLA-G genotype, suggesting a more innate character of the interacting receptors for HLA-G. Taken together, this work demonstrates (i) a clear distinction in binding efficiency of the three allelic HLA-G subtypes and (ii) a significant difference in HLA-G allele–mediated proliferation of primary NK cells from human term placenta. In the light of recent studies that tried to generate dNK cells in vitro (Cerdeira et al. 2013) and findings that the sHLA-G level in embryo culture medium correlates with successful pregnancy (Lynge Nilsson et al. 2014), with the overall goal to treat reproductive disorders, this investigation could support those efforts by recommending the allelic variant HLA-G*01:04 for further use to develop a most efficient treatment.

This work shows the broad functional variability of HLA-G subtypes and implies the need for HLA-G genotyping. Many cellular therapeutic strategies could be supported by the use of synthetic HLA-G and/or by the knowledge about the HLA-G genotype of the patient. Furthermore, the knowledge on HLA-G and its biological function appears to be crucial to understand and cure reproductive disorders.

## Electronic supplementary material

ESM 1 (PDF 204 kb)

ESM 2 (PDF 62 kb)
