# Supplementary material for: The microstructure in the placenta is influenced by the functional diversity of HLA-G allelic variants
Source: Immunogenetics. 2019 Jun 27;71(7):455–63. doi: 10.1007/s00251-019-01121-0 (PMC6647172; doi:10.1007/s00251-019-01121-0)
Supplement: Supplementary file 1 — (PDF 204 kb) [file 251_2019_1121_MOESM1_ESM.pdf]

Supplemental Fig. 1: Binding of CD56$^{+bright}$/CD9^{-} NK cells to sHLA-G*01:01/01:03 or 01:04

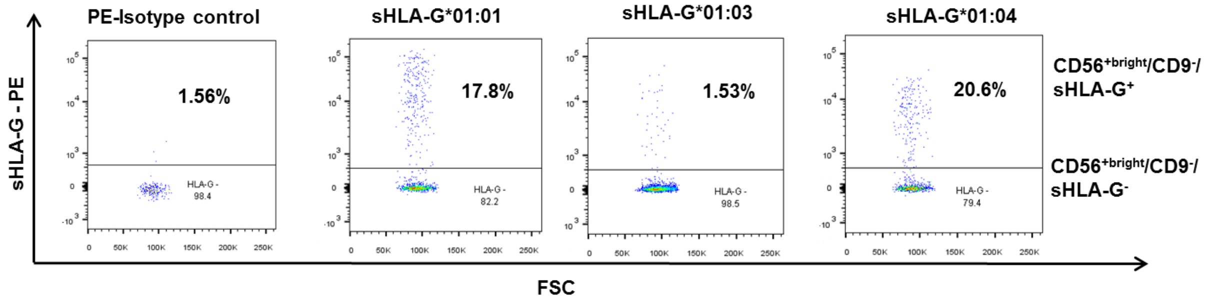

Analysis of sHLA-G binding to the non-dNK population (CD56$^{+bright}$/CD9^{-}) from term placenta.
