# Supplementary material for: The microstructure in the placenta is influenced by the functional diversity of HLA-G allelic variants
Source: Immunogenetics. 2019 Jun 27;71(7):455–63. doi: 10.1007/s00251-019-01121-0 (PMC6647172; doi:10.1007/s00251-019-01121-0)
Supplement: Supplementary file 2 — (PDF 62 kb) [file 251_2019_1121_MOESM2_ESM.pdf]

Supplemental Fig. 2: Proliferation of CD56$^{+bright/}$CD9^{-} NK cells after exposure to different HLA-G allelic variants

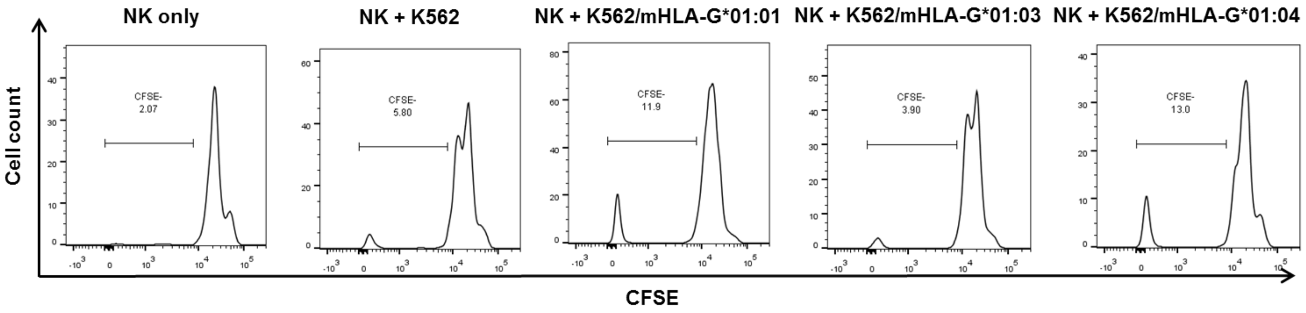

Analysis of proliferation of the primary non-dNK cell population (CD56$^{+bright}$/CD9^{-}) from term placenta after co-incubation with HLA-G*01:01/01:03 or 01:04 expressing *K562* cells.
